# Antigen-induced arthritis in rats is associated with increased growth-associated protein 43–positive intraepidermal nerve fibres remote from the joint

**DOI:** 10.1186/s13075-015-0818-8

**Published:** 2015-10-26

**Authors:** Elisabeth Jochmann, Michael Karl Boettger, Praveen Anand, Hans-Georg Schaible

**Affiliations:** Institute of Physiology 1/Neurophysiology, Jena University Hospital, Friedrich Schiller University, Teichgraben 8, 07743 Jena, Germany; Peripheral Neuropathy Unit, Imperial College London, Hammersmith Hospital Campus, Du Cane Road, London, W12 0NN UK; Present address: Bayer HealthCare AG, 42096 Wuppertal, Germany

**Keywords:** Antigen-induced arthritis, Rat, Growth-associated protein 43, Intraepidermal nerve fibres

## Abstract

**Introduction:**

Pain in arthritis may be experienced in regions outside the affected joint, and hyperalgesia may even be widespread. The spread of pain is usually attributed to mechanisms in the central nervous system. We investigated whether rats with antigen-induced arthritis (AIA) exhibit peripheral changes in skin innervation remote from the inflamed joint.

**Methods:**

After immunization, unilateral AIA in the knee joint was induced in rats. Intraepidermal nerve fibre density was determined by immunohistochemical staining for protein gene product 9.5 (PGP 9.5) and for nerve fibres expressing calcitonin gene–related peptide (CGRP), substance P (SP), transient receptor potential vanilloid 1 (TRPV1; the heat and capsaicin receptor), β-tubulin, and growth-associated protein 43 (GAP-43; a marker of regenerating nerve fibres) in paw pad skin and back skin. Cluster of differentiation 11b (CD11b)-positive macrophages and CD3-positive T cells were quantified in skin, and macrophages were quantified in the lumbar dorsal root ganglia. In addition, pain-related behaviour was assessed.

**Results:**

Intraepidermal nerve fibre density (PGP 9.5) and the numbers of fibres expressing CGRP, SP, TRPV1, or β-tubulin did not show a significant change in the acute (3 days) or chronic phase (21 days) of AIA compared with control rats that were only immunized. However, paw skin and back skin revealed a significantly higher number of nerve fibres expressing GAP-43 at both the acute and chronic stages of AIA. The skin of arthritic rats in these regions did not contain a greater density of CD11b and CD3 immune cells than the skin of control rats. Enhanced expression of GAP-43 in nerve fibres of the skin was not related to hyperalgesia in the joint, but it accompanied persistent secondary cutaneous hyperalgesia in the skin remote from the inflamed joint.

**Conclusions:**

Although the innervation of the skin remote from the joint did not show significant abnormalities of the other nerve fibre markers, the rapid and persistent increase of GAP-43 expression is conspicuous. The data suggest that immune-mediated arthritis is associated with changes in skin innervation remote from the inflamed joint, although the skin is not inflamed, which may contribute to symptoms in nonarticular tissue remote from the affected joint.

## Introduction

Chronic joint diseases such as rheumatoid arthritis and osteoarthritis are usually associated with chronic pain in the affected joints. Careful clinical examination has shown, however, that pain is often not restricted to the affected joints. Patients with localized osteoarthritis and rheumatoid arthritis often exhibit allodynia (a reduction of pain thresholds in the innocuous intensity range) and hyperalgesia (increased pain perception on noxious stimulation) in the entire limb [1, 2]. These phenomena are reproduced in experimental arthritis models such as antigen-induced arthritis (AIA) [3, 4]. Research in humans and experimental models suggests that the spread of pain is due to neuronal mechanisms in the central nociceptive system, such as the sensitization of spinal cord neurons with expansion of receptive fields (central sensitization) [5, 6], sensitization of cortical pain–processing areas [7], reduced activity of endogenous inhibitory systems descending from the brainstem, and others [8, 9]. Such changes may initially be induced by enhanced nociceptive inputs from sensitized peripheral nociceptors [10, 11].

However, pain states are not entirely characterized by augmented functional activity in the peripheral and central nociceptive systems. An intriguing finding is the change of the innervation density of diseased joints. Inflamed synovial tissue derived from experimental arthritis and from patients with rheumatoid arthritis showed a significant reduction of nerve fibre density. Some authors reported a reduction of peptidergic sensory afferents [12–16], whereas others described mainly a reduction of sympathetic nerve fibres [17]. A recent study reported a significant reduction of nerve fibres in the synovium of patients with osteoarthritis [18], but areas of sprouting of nerve fibres were also reported in affected joints [19].

Changes of innervation density are likely to be caused by local factors in the joint. However, to our knowledge, whether changes of the innervation density in joint diseases are restricted to the affected joint, or whether such changes also occur remote to the afflicted joint, has never been systematically tested. We therefore addressed this question in the present study using the AIA model in the rat, an animal model of monoarticular inflammation usually induced in the knee joint, which reproduces major aspects of clinical rheumatoid arthritis. Rats with AIA show a highly reproducible pathology with well-defined acute and chronic states. Pronounced inflammation of the affected knee joint with local swelling and hypersensitivity, as well as hyperalgesia in areas remote from the inflammatory process (e.g., the noninflamed contralateral knee and the paws), have been described [3, 20]. Specifically, we asked whether the density of intraepidermal nerve fibres (IENFs) is altered in the paws and the back skin (regions far from the knee joint). To investigate this question, we performed staining for protein gene product 9.5 (PGP 9.5), a structural nerve marker. We also quantified the IENFs expressing the neuropeptides calcitonin gene–related peptide (CGRP) and substance P (SP), both peptides are released from nerve fibres and contribute to neurogenic inflammation; the ion channel transient receptor potential vanilloid 1 (TRPV1), the heat and capsaicin receptor [21]; and the cytoskeletal protein β-tubulin. Furthermore, we studied growth-associated protein 43 (GAP-43), which is associated with nerve regeneration [22]. In order to identify inflammatory changes in the skin, we labelled skin sections for cluster of differentiation 11b (CD11b) (macrophages) and CD3 (T cells). We found that the paw pad skin and the back skin of rats with AIA showed stable numbers of PGP 9.5–positive IENFs and that the number of CGRP-positive, SP-positive, TRPV1-positive, and β-tubulin–positive IENFs in the paw pad skin also stayed stable, all in the absence of inflammatory signs. Interestingly, however, the AIA rats exhibited significantly enhanced numbers of GAP-43–positive IENFs in both the acute and chronic stages of AIA.

## Methods

### Animals

The study on AIA was performed on 25 female Lewis rats (age 6–8 weeks initially, 160–190 g; Charles River Laboratories, Sulzfeld, Germany). All experiments were carried out in accordance with the European Communities Council Directive (86/609/EEC) for the care and use of laboratory animals and were approved by the Thuringian State Office of Food Safety and Consumer Protection, Department of Consumer Protection, Veterinary, Pharmaceuticals (registration number 02-013/08).

### Antigen-induced arthritis

Arthritis was induced as previously described [3]. In brief, with an interval of 7 days, rats (*n* = 25) were immunized by two subcutaneous injections of 500 μg of methylated bovine serum albumin (mBSA; Sigma, Deisenhofen, Germany) prepared in saline, emulsified with 500 μl of complete Freund’s adjuvant (Sigma) supplemented with 2 mg/ml *Mycobacterium tuberculosis* strain H37Ra (Difco; Becton Dickinson, Sparks, MD, USA). Fourteen days after the second injection, monoarticular arthritis was induced by a further injection of mBSA (500 μg in 50 μl of saline) into the left knee joint cavity (*n* = 20). The five immunized-only rats served as controls.

Either at 3 days (*n* = 10, acute AIA) or at 21 days (*n* = 10, chronic AIA) after induction of AIA, the rats were anesthetized by intraperitoneal injection of sodium thiopentone (120 mg/kg Trapanal; Byk Gulden, Konstanz, Germany) and killed by transcardial perfusion with heparin-enriched, phosphate-buffered saline (PBS) and 4 % phosphate-buffered formalin.

### Assessment of swelling and pain-related behaviour

For the assessment of swelling of the knee joint, the mediolateral diameter of each knee was measured with a vernier calliper (Mitutoyo, Neuss, Germany) as described previously [3]. The difference between the diameter of the noninflamed and inflamed knees for each animal and testing day indicates the relative swelling.

Using an incapacitance tester (Linton Instrumentation, Norfolk, UK), weight-bearing of the inflamed hind limb was assessed as a functional measure of pain-related guarding behaviour. The animals were placed with their hind paws on scales inside a plastic cage, and the weight force on the two scales was measured three consecutive times while the animals sat calmly in the cage after acclimation. From these averaged values, the relative resting weight (expressed as a percentage) of the inflamed hind limb was evaluated [weight of inflamed hind limb × 100 %/(weight of inflamed hind limb + weight of noninflamed hind limb)], as described previously [23].

Primary hyperalgesia at the inflamed knee was assessed with a dynamometer (Correx, Bern, Switzerland) [24]. At the level of the joint space on the lateral side of the knee joint, increasing pressure was applied with the dynamometer until the animals attempted to escape or vocalized. The applied weight force was read in grams. A cutoff value was defined at 250 g to prevent tissue damage. Testing was performed once for each animal on each testing day to avoid nociceptive sensitization due to repeated testing.

As previously described [3, 4], secondary hyperalgesia at the paw was evaluated by testing paw withdrawal thresholds to mechanical stimulation using a dynamic plantar aesthesiometer (Ugo Basile, Comerio, Italy). For this test, the animal rested on a mesh floor and a blunt filament touched the hind paw on the plantar surface with increasing force until the animal withdrew its leg. The weight force needed for this reaction was measured in grams. After habituation to the device (15 min), three measurements were recorded and the mean was used as the mechanical hyperalgesia threshold value. Animals were tested before induction of AIA (baseline) and on days 3 (acute AIA), 7, 14, and 21 (chronic AIA) after induction of AIA. All pain-related tests were performed on ten acute AIA animals and ten chronic AIA animals, except for the assessment for secondary hyperalgesia, which was performed on five animals per group.

### Tissue processing and immunostaining

After the animals were killed, hind paw pad skin (five immunized controls, ten ipsilateral acute and chronic AIA rats, and five contralateral acute and chronic AIA rats), lumbar paraspinal back skin (five immunized controls and ten contralateral acute and chronic AIA rats), and lumbar dorsal root ganglia (DRGs) from spinal levels L1–L5 (ten ipsilateral acute AIA, nine ipsilateral chronic AIA, five contralateral acute AIA, and five contralateral chronic AIA rats) were dissected separately. The specimens were then processed and immunostained as described previously [25]. Briefly, AIA tissues were immersed in Zamboni’s fixative (2 % wt/vol formalin, 0.1 M phosphate, 15 % vol/vol saturated picric acid) for 1 h at room temperature and then stored in PBS containing sucrose and sodium azide at 4 °C. The optimally orientated specimens were embedded in optimal cutting temperature compound (Tissue-Tek OCT; RA Lamb, Eastbourne, UK) and snap-frozen. Frozen specimens were sectioned (30-μm and 15-μm thicknesses) using a cryostat (Bright Instruments, Huntingdon, UK), and sections were collected on precoated glass slides (VWR International, Lutterworth, UK). Sections were then air-dried, and endogenous peroxidase was blocked by incubating the sections in industrial methylated spirits containing 0.3 % vol/vol hydrogen peroxide for 30 min. After rehydration to PBS, the sections were incubated overnight at 4 °C with primary antibodies to the structural nerve marker PGP 9.5 (1:40,000, 30-μm sections, paw pad skin and back skin; UltraClone Ltd, Wellow, UK), the cytoskeletal protein β-tubulin (1:2000, 30-μm sections, paw pad skin only; R&D Systems, Minneapolis, MN, USA), the neuropeptides CGRP (1:4000, 30-μm sections, paw pad skin only; Chemicon International, Chandler’s Ford, UK) and SP (1:2000, 30-μm sections, paw pad skin only; Chemicon International), the ion channel TRPV1 (1:200, 30-μm sections, paw pad skin only; GlaxoSmithKline, Harlow, UK), and GAP-43 (1:2000, 30-µm sections, paw pad skin: Dr. G.P. Wilkin, Imperial College London, London, UK; 1:20,000, 30-μm sections, back skin: Sigma-Aldrich, Gillingham, UK) [26]. Different primary antibodies to GAP-43 were applied for the two types of skin owing to the better quality of staining achieved with the correspondent antibody, which could be explained by structural differences in glabrous and hairy skin. Furthermore, primary antibodies to inflammatory cell markers CD11b for macrophages (1:100, 15-μm sections, paw pad skin only; AbD Serotec, Kidlington, UK), CD3 for T cells (1:200, 15-μm sections, paw pad skin only; Abcam, Cambridge, UK), and ED1 (CD68) for macrophages (1:200, 15-μm sections, DRGs; AbD Serotec) were used. Thicker (30-μm) sections were used to increase the sensitivity for markers of nerves, particularly fine, penetrating IENFs, whereas thinner (15-μm) sections were optimal for cells detected with inflammatory cell markers. Sites of primary antibody attachment were revealed using a standard nickel-enhanced immunoperoxidase method, providing a black immunopositive reaction product [27]. The sections were counterstained for nuclei with 0.1 % wt/vol aqueous neutral red and mounted in a xylene-based mountant before analysis. Control experiments were performed by omission of the primary antibodies.

### Quantification of immunostaining

IENFs immunoreactive to PGP 9.5, CGRP, SP, β-tubulin, and GAP-43 were counted in a blinded manner along the length of the tissue section (approximately 3–8 mm) according to the guidelines developed by the European Federation of Neurological Societies (EFNS) [28]. Thus, only nerve fibres visibly crossing the basement membrane of the epidermis were counted. For assessment of whether a fibre crossed the basement membrane, the focus of the microscope was adjusted to follow the nerve fibre’s course within the dermis and epidermis. Nerve fibres that branched in the epidermis were counted as one; nerves branching below or within the basement membrane were counted as separate units. Nerve fragments within the epidermis were not counted [28]. Three nonadjacent sections from each animal were analysed for PGP 9.5; a further three separate sections for GAP-43 (for paw pad and back skin); and a single section for CGRP, SP, and β-tubulin (for paw pad skin only). The length of the epidermis was measured on images captured using an Olympus DP70 camera mounted on an Olympus BX50 microscope using Olympus analySIS software (version 5.0 for Windows; Olympus, Hamburg, Germany). The results are expressed as nerve fibres per millimetre length of epidermis.

CD11b and CD3 immunoreactivity in the dermis and ED1 immunoreactivity in the DRGs were quantified by computerized image analysis. Images were captured using an Olympus DP70 camera mounted on an Olympus BX50 microscope and analysed using Olympus analySIS software. After applying the colour separation for red to blank out the counterstaining, positive immunostaining was highlighted by setting the grey-level detection limit to a fixed threshold, and the area of highlighted immunoreactivity obtained as a percentage of the field scanned. The threshold was kept constant for all specimens analysed that were immunostained with the same marker. For the skin (one section per animal) and DRGs (three nonadjacent sections per animal) five and three microscopic fields (magnification × 40) were analysed, respectively, and the mean values were used in statistical analysis.

### Statistical analysis

Descriptive statistics were created and statistical tests were performed using Prism version 5.03 for Windows software (GraphPad Software, San Diego, CA, USA). Group differences for the immunostaining data were assessed using the nonparametric Mann–Whitney *U* test (two-tailed). Behavioural changes were examined with the Wilcoxon matched-pairs signed-rank test (two-tailed) and intra- and interobserver correlations as well as IENF correlations with Spearman’s correlation coefficient. Significance was assumed at *p* < 0.05.

## Results

### Knee swelling and pain-related behaviour

**Fig. 1:**
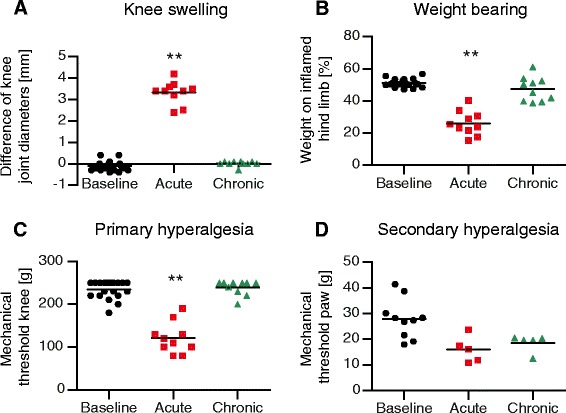
Joint swelling and pain-related behaviour. **a** The ipsilateral knee joint showed pronounced swelling in the acute state of antigen-induced arthritis (AIA) compared with baseline values obtained before induction of AIA. **b** Animals with acute AIA bore significantly less weight on the inflamed hind limb compared with baseline values. **c** Withdrawal thresholds for mechanical stimulation of the knee joint were significantly decreased in the acute state of AIA. **d** Withdrawal thresholds for monofilament stimulation of the paw pad decreased in animals with AIA, but the values did not reach statistical significance. Baseline values for acute and chronic AIA were pooled for the graphs, as there were no significant differences between the values in these two groups. **p < 0.01

Injection of mBSA into the knee joints of immunized rats caused unilateral arthritis. Swelling of the injected knee was pronounced during the first 3 days and decreased nearly to the prearthritic baseline until day 21. The difference in knee joint diameters was significantly increased (*p* = 0.0059) for the acute AIA vs. baseline values (Fig. 1a).

Animals with AIA showed asymmetric weight-bearing, putting significantly more weight on the noninjected joint in the acute state compared with baseline values (*p* = 0.002). This pain-related guarding behaviour subsided gradually by day 21 (chronic state) (Fig. 1b).

Rats withdrew their legs or vocalized when pressure was applied onto the knee. The mechanical pain thresholds on the knee were significantly decreased in the acute state of AIA compared with baseline values (*p* = 0.0058), demonstrating primary hyperalgesia at the inflamed knee. By day 21, the mechanical thresholds had increased again to values close to baseline (Fig. 1c).

Withdrawal thresholds for monofilament stimulation of the hind paw pad in rats with AIA were lower than, but not significantly different from, baseline values (acute vs. baseline *p* = 0.0625, chronic vs. baseline *p* = 0.125, *n* = 5 for acute AIA and chronic AIA groups). This indicates a trend towards secondary hyperalgesia remote from the inflammation (Fig. 1d).

### Intraepidermal nerve fibres in paw pad and back skin

**Fig. 2:**
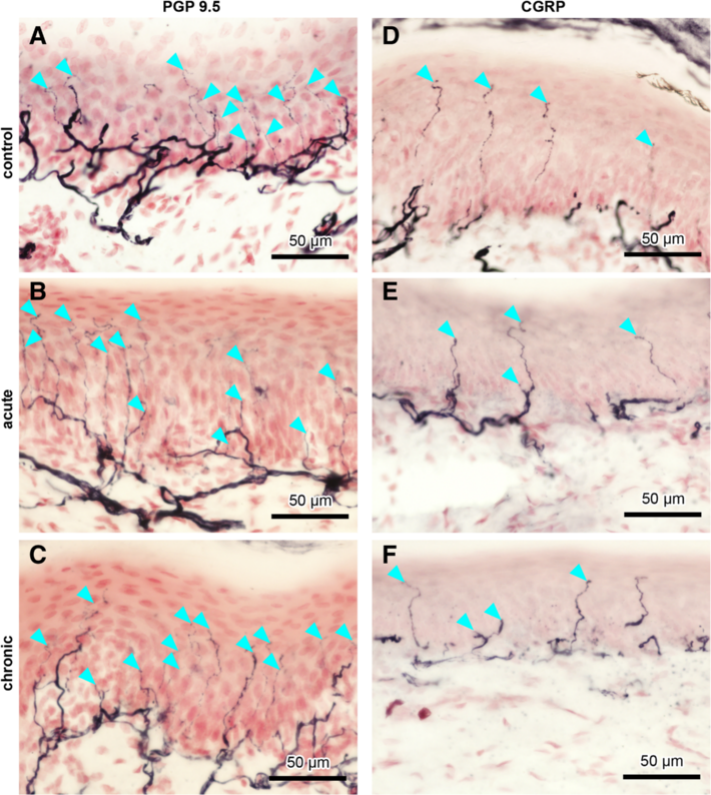
Intraepidermal nerve fibres (IENFs) in ipsilateral rat paw pad skin. Nerve fibres immunopositive for protein gene product 9.5 (PGP 9.5) (**a**–**c**) and calcitonin gene–related peptide (CGRP) (**d**–**f**) in controls (**a**, **d**), acute AIA (**b**, **e**), and chronic AIA rats (**c**, **f**). *Turquoise arrowheads* indicate fine-calibre intraepidermal nerve fibres that were counted according to the European Federation of Neurological Societies' counting rules, thus visibly crossing the basement membrane between epidermis and dermis. For evaluation of IENF density, the focus of the microscope was adjusted while we analysed the individual sections. Therefore, not all IENFs that are marked by *turquoise arrowheads* can be followed all the way through the epidermis in the images. Thicker fibres along the dermal–epidermal junction are subepidermal nerve fibres. Numbers of IENFs that reacted with antibodies against PGP 9.5 and CGRP did not change in animals with AIA compared with immunized-only controls. Original magnification × 40

In rat hind paw pads and back skin, intense PGP 9.5–immunoreactive subepidermal nerve fibres of various calibres were present along the length of the dermal–epidermal junction. Many fine-calibre nerve fibres penetrated into the epidermis (IENFs), some extending almost to the stratum corneum (Fig. 2a–c, ipsilateral paw pad skin). In paw pad skin, a similar pattern was seen for CGRP-immunoreactive nerve fibres, but with clearly fewer fibres overall (Fig. 2d–f, ipsilateral paw pad skin), as well as for SP-, TRPV1-, and β-tubulin–positive fibres. These observations applied to all samples from the three groups examined (immunized controls, acute AIA, and chronic AIA), with no obvious differences in numbers of nerve fibres between groups for either marker.

**Fig. 3:**
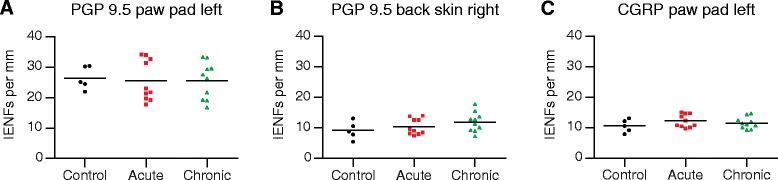
Measurement of intraepidermal nerve fibres (IENFs) in the paw pad and back skin. Numbers of IENFs per millimetre in control rats, acute antigen-induced arthritis (AIA) rats, and chronic AIA rats, which are immunopositive for protein gene product 9.5 (PGP 9.5) (**a**, **b**) and calcitonin gene–related peptide (CGRP) (**c**) in the paw pad (**a**, **c**) and back skin (**b**). Counts of IENFs immunopositive to PGP 9.5 and CGRP did not show significant changes

Counts of IENFs per millimetre length of epidermis of AIA rats remained at about the same level as in controls in sections immunostained with antibodies to PGP 9.5, CGRP (Fig. 3a and c, ipsilateral paw pad; Fig. 3b, contralateral back skin), SP, TRPV1, and β-tubulin. The numbers of IENFs that were positive for PGP 9.5 are represented in Table 1, numbers of IENFs that were positive for CGRP, SP, TRPV1, or β-tubulin are represented in Table 2.

**Table 1:** Numbers of protein-gene product 9.5 (PGP 9.5)–positive intraepidermal nerve fibres (IENFs)

	Controls	Acute AIA	Chronic AIA	Average SD
Ipsilateral paw pad skin	26.42 ± 3.75	25.51 ± 6.71	25.60 ± 6.02	5.27
Back skin	9.14 ± 2.87	10.39 ± 2.57	11.89 ± 3.20	2.86

Numbers of IENFs per mm epidermis that were positive for PGP 9.5 in the left (ipsilateral) paw pad skin and in the lumbar paraspinal back skin are presented as mean ± standard deviation. The average SD is the average standard deviation between counts of the three evaluated sections per animal. There were no significant changes in counts of IENFs for stainings with PGP 9.5

**Table 2:** Numbers of intraepidermal nerve fibres in paw pad skin for other nerve fibre markers

	Controls	Acute AIA	Chronic AIA
CGRP	10.57 ± 2.13	12.21 ± 2.12	11.50 ± 1.88
SP	5.38 ± 1.05	4.87 ± 2.17	5.27 ± 1.30
TRPV1	7.17 ± 2.44	6.92 ± 5.41	6.46 ± 4.65
β-tubulin	26.63 ± 7.69	23.58 ± 6.54	22.34 ± 3.89

Numbers of IENFs per mm length of epidermis that were positive for calcitonin gene-related peptide (CGRP), substance P (SP), transient receptor potential vanilloid 1 (TRPV1), and β-tubulin in left (ipsilateral) paw pad skin are presented as mean ± standard deviation. There were no significant changes in counts of IENFs for these nerve fibre markers

**Fig. 4:**
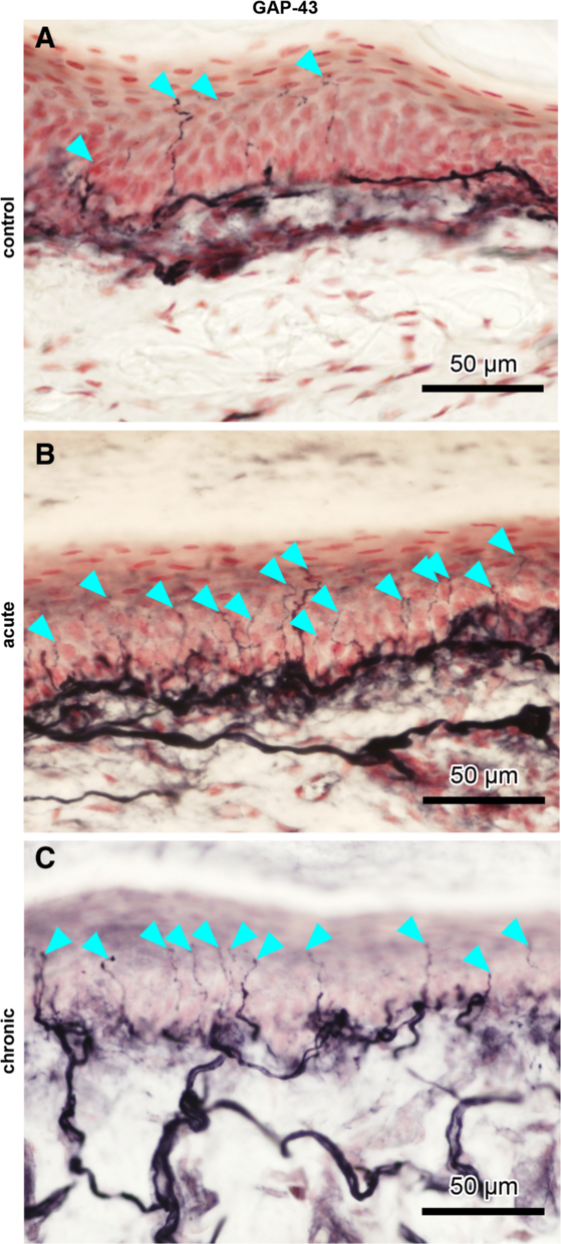
Intraepidermal nerve fibres (IENFs) in ipsilateral rat paw pad skin. Nerve fibres immunopositive for growth-associated protein 43 (GAP-43) in control rats (**a**), acute antigen-induced arthritis (AIA) rats (**b**), and chronic AIA rats (**c**). *Turquoise arrowheads* indicate fine-calibre IENFs that were counted according to the European Federation of Neurological Societies' counting rules, thus visibly crossing the basement membrane between epidermis and dermis. For evaluation of IENF density, the focus of the microscope was adjusted while we analysed the individual sections. Therefore, not all IENFs that are marked by *turquoise arrowheads* can be followed all the way through the epidermis in the images. Thicker fibres along the dermal–epidermal junction are subepidermal nerve fibres. Numbers of IENFs that reacted with antibodies against GAP-43 increased significantly after induction of AIA. Original magnification × 40

Skin sections immunostained with the antibody to GAP-43 also revealed intense staining of subepidermal nerve fibres. Control animals showed only few labelled IENFs (Fig. 4a), but apparently more IENFs were found in acute or chronic AIA rats (Fig. 4b and c, ipsilateral paw pad skin). The same observations were made for back skin sections.

**Fig. 5:**
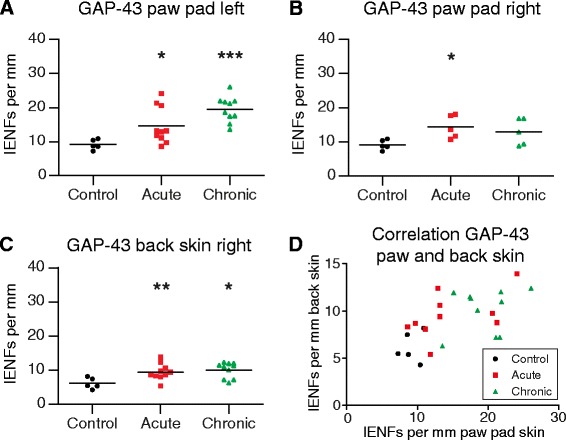
Growth-associated protein 43 (GAP-43)–positive intraepidermal nerve fibres (IENFs) in paw and back skin and their correlation. Numbers of IENFs per millimetre epidermis immunopositive for GAP-43 in control rats, acute antigen-induced arthritis (AIA) rats, and chronic AIA rats (**a**–**c**). Numbers of IENFs marked by antibodies against GAP-43 increased significantly in the left paw pad skin (ipsilateral) and in lumbar back skin from paraspinal right (contralateral) in the acute and chronic state of AIA, compared with immunized-only controls (**a**, **c**). GAP-43–positive IENFs were significantly increased in the right paw pad skin (contralateral) in the acute state of AIA but not in the chronic state (**b**). Numbers of IENFs positive for GAP-43 per millimetre of left paw pad skin correlated to those of lumbar back skin (**d**). *R* = 0.6023, *p* = 0.0014; *p < 0.05, **p < 0.01, ***p < 0.001

Counts of GAP-43–immunoreactive IENFs were increased significantly in paw pad skin from rats with acute (*p* = 0.0127) and chronic (*p* = 0.0007) AIA as compared with controls (Fig. 5a, ipsilateral). The same applied for numbers of GAP-43–positive IENFs in the contralateral back skin (acute AIA vs. controls *p* = 0.0080, chronic AIA vs. controls *p* = 0.0193; Fig. 5c). These changes in GAP-43 counts also occurred in the contralateral paw (*n* = 5) with the increase of GAP-43 being significant in acute AIA (*p* = 0.0159), but not in chronic AIA (*p* = 0.0952), compared with controls (Fig. 5b). The numbers of IENFs that were positive for GAP-43 are represented in Table 3. The numbers of GAP-43–immunoreactive IENFs per millimetre paw pad skin and per millimetre back skin correlated positively (*R* = 0.6023, *p* = 0.0014; Fig. 5d).

**Table 3:** Numbers of growth-associated protein 43 (GAP-43)–positive intraepidermal nerve fibres (IENFs)

	Controls	Acute AIA	Chronic AIA	Average SD
Ipsilateral paw pad skin	9.16 ± 1.47	14.65 ± 5.36	19.48 ± 3.73	5.14
	*Significance of the group difference against the control group:*	*p = 0.0127*	*p = 0.0007*	
Contralateral paw pad skin	9.16 ± 1.47	14.34 ± 3.39	12.95 ± 3.89	4.13
	*Significance of the group difference against the control group:*	*p = 0.0159*	*p = 0.0952*	
Back skin	6.17 ± 1.62	9.54 ± 2.37	10.10 ± 2.31	2.68
	*Significance of the group difference against the control group:*	*p = 0.0080*	*p = 0.0193*	

Numbers of IENFs per millimetre epidermis that were positive for GAP-43 in the left (ipsilateral) and right (contralateral) paw pad skin and in the lumbar paraspinal back skin are presented as mean ± standard deviation. The average SD is the average standard deviation between counts of the three evaluated sections per animal. Significances of the group differences were assessed using the nonparametric Mann Whitney test

Intra- and interobserver agreements for the counts of IENFs were highly significant, as verified by correlations (intraobserver reliability for paw pad skin tested on two counts each for ten randomly chosen slides: *R* = 0.9879, *p* < 0.0001; interobserver reliability for paw pad skin tested on one count by two observers for ten randomly chosen slides: *R* = 0.8303, *p* = 0.0047).

### Relationship between behavioural data and GAP-43–positive IENFs

**Fig. 6:**
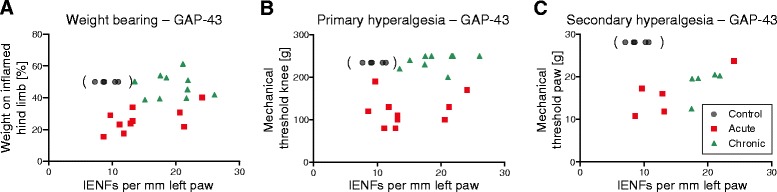
Plots for growth-associated protein 43 (GAP-43)–positive intraepidermal nerve fibres (IENFs) in paw skin and behavioural data of antigen-induced arthritis (AIA) rats. **a** Relationship between the relative weight-bearing on the inflamed hind limb and IENFs marked by antibodies against GAP-43 in the ipsilateral paw pad skin. Relative weight-bearing for control animals was defined as 50 %, with equal weight on both legs. Control values are parenthesized. **b** Plot of the mechanical threshold at the inflamed knee and GAP-43–positive IENFs in the ipsilateral paw pad skin. Control values in parentheses were set at 234.5 g, which was the average baseline value for animals before induction of AIA (standard deviation was ±20.6 g). **c** Relationship between the mechanical threshold of the ipsilateral paw pad and GAP-43–positive IENFs in the ipsilateral paw pad skin. Control values in parentheses were determined as the mean of baseline values from acute and chronic AIA animals before induction of AIA (27.9 g ± 7.6 g)

In further analyses, we asked whether the number of GAP-43–positive IENFs is correlated to different parameters in assessment of pain-related behaviour. Figure 6 shows plots of different pain parameters and GAP-43–positive IENFs in the skin of the left paw of individual animals in the acute and chronic AIA groups. Because all the rats that were tested for nociceptive behaviour before induction of AIA were used for the induction of AIA (see Fig. 1), we used another five immunized control rats to determine the baseline of GAP-43–positive IENFs. These animals did not undergo behavioural testing. For the display of these data in Fig. 6, we therefore used the typical range of the values of behavioural parameters before induction of AIA (see Fig. 1). The baseline data of Fig. 1 correspond to values of our previous studies [3, 4, 29] [i.e., the weight-bearing is symmetric, the mechanical threshold at the noninflamed knee is slightly below 250 g (250 g is the cutoff value, average of baseline values here 234.5 g ± 20.6 g), and the mechanical threshold at the paw is around 30 g (average here 27.9 g ± 7.6 g)].

At the acute stage of AIA, in which the values of the pain parameters indicated pronounced hyperalgesia, the values of GAP-43–positive IENFs were scattered, extending from the normal range to distinct increases (Fig. 6a – c). However, at the chronic stage of AIA, the animals showed a strong reversal of weight-bearing and primary hyperalgesia, but all rats exhibited an increase of GAP-43–positive IENFs (Fig. 6a and b). Secondary hyperalgesia at the ipsilateral paw remained visible (Fig. 6c). These data suggest that the change of GAP-43 expression is a dynamic process starting at the acute stage of AIA. However, they do not indicate a simple, straight association between GAP-43 expression and pain.

### Inflammatory cell markers in paw pad skin and DRGs

**Fig. 7:**
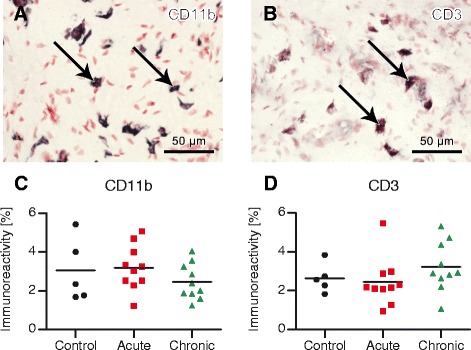
Inflammatory cell markers in ipsilateral rat paw pads. Cells in the dermis and image analysis (% area) of cluster of differentiation 11b (CD11b) immunoreactivity (**a**, **c**; macrophages) and CD3 immunoreactivity (**b**, **d**; T cells) in control and antigen-induced arthritis (AIA) rats. *Arrows* mark typical CD11b-positive (**a**) and CD3-positive (**b**) cells. There was no significant change of inflammatory cells in paw pad skin of AIA rats compared with controls. Original magnification × 40

Throughout the dermis of the ipsilateral rat paw pad CD11b-immunoreactive (macrophages) and CD3-immunoreactive (T cells) cells appeared to be of similar density within the three groups examined (Fig. 7a and b). This was confirmed by image analysis of CD11b and CD3 staining, which did not reveal any significant differences (Fig. 7c and d). This indicates that there was no inflammatory process in the paw pad remote from the inflamed knee joint.

**Fig. 8:**
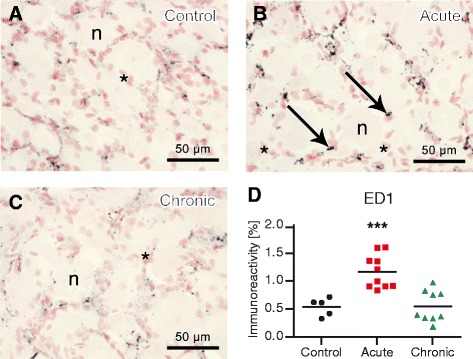
Macrophages in ipsilateral rat lumbar dorsal root ganglia (DRGs). Anti-CD68 antibody (ED1) immunoreactivity in control rats (**a**), acute antigen-induced arthritis (AIA) rats (**b**), and chronic AIA rats (**c**) and image analysis (% area) of ED1 immunoreactivity (**d**). There were significantly more macrophages in the DRGs of animals with acute AIA compared with controls. *Arrows* mark typical ED1-positive cells. *Satellite cells, *n* = neurons. Original magnification × 40. ***p < 0.001

ED1-immunoreactive cells (macrophages) were detected around sensory neuronal perikarya of lumbar DRGs (Fig. 8a – c). They appeared to be more numerous in acute AIA rats than in control and chronic AIA animals in both ipsi- and contralateral DRGs. Image analysis of ED1 immunostaining showed a highly significant increase (*p* = 0.0007) in acute AIA animals compared with controls on the ipsilateral side (Fig. 8d) and on the contralateral side (*p* = 0.0079).

### Relationship between ED1 immunoreactivity in lumbar DRGs and GAP-43-positive IENFs as well as ED1 and behavioural data

**Fig. 9:**
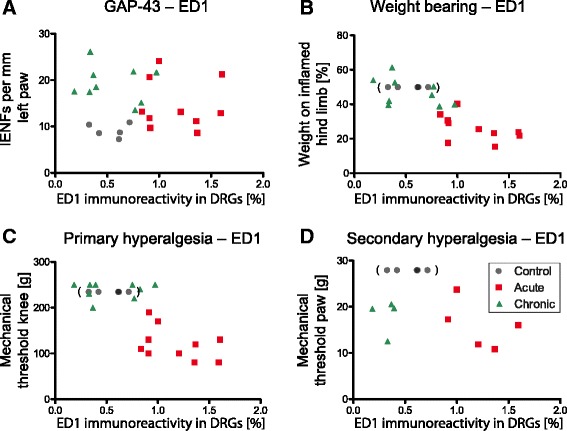
Anti-CD68 antibody (ED1) immunoreactivity in lumbar dorsal root ganglia (DRGs) and its correlations with growth-associated protein 43 (GAP-43)–positive intraepidermal nerve fibres (IENFs) and behavioural data. Plot of the time course of GAP-43–positive IENFs in the left paw pad skin and ED1 immunoreactivity (macrophage invasion) in the lumbar DRGs (**a**). Correlation between ED1-immunoreactive cells in the DRGs and weight-bearing. Relative weight bearing for control animals was defined as 50 %, with equal weight on both legs. Control values are parenthesized (**b**). Correlation between ED1-immunoreactive cells in the DRGs and primary hyperalgesia. Control values in parentheses were set at 234.5 g, which was the average baseline value from animals before induction of AIA (SD was ±20.6 g) (**c**). Relationship of ED1 immunoreactivity and secondary hyperalgesia in the paw. Control values in parentheses were determined as the mean of baseline values from acute and chronic AIA animals before induction of AIA (27.9 g ± 7.6 g) (**d**)

We next examined potential correlations between ED1 in the lumbar DRGs and GAP-43–positive IENFs in the paw pad and between ED1 and pain-related behaviour. Figure 9a demonstrates the increased ED1 immunoreactivity and therewith invasion of macrophages into the lumbar DRGs in the acute phase of AIA and its decline back to the level of control values in the chronic phase. Numbers of GAP-43–positive IENFs started to increase in the acute stage of AIA and were strongly elevated in the chronic stage. The plot in Fig. 9 clearly depicts a lack of strict correlation between macrophages in the DRGs and the density of GAP-43–positive IENFs at this time point.

However, the invasion of macrophages into the DRGs for acute and chronic AIA rats was correlated with weight-bearing (*R* = −0.7930, *p* < 0.0001) (Fig. 9b) and with primary hyperalgesia of the knee (*R* = −0.7116, *p* = 0.0006) (Fig. 9c). Control values were not included in this analysis, as control animals did not undergo behavioural testing, as mentioned above (see Fig. 6). Nevertheless, the averaged baseline values of acute and chronic AIA animals before induction of AIA that were used for the plot to depict the potential position of control values seemed to lie within the area that would be expected for overall correlation. In contrast, ED1 in DRGs and secondary hyperalgesia in the paw were not strictly correlated, as the decrease of the pain threshold persisted and did not fully reverse in the chronic phase of AIA (Fig. 9d). This underlines the existence of different mechanisms for the development of primary hyperalgesia in the inflamed region and secondary hyperalgesia remote from the affected joint.

## Discussion

In the present study, we investigated whether localized arthritis in the knee joint causes neuronal changes in the skin remote from the joint. Neither overall IENF density nor the numbers of fibres expressing CGRP, SP, TRPV1, or β-tubulin showed a significant change in the acute or chronic phase of AIA compared with control rats. In contrast, the paw and back skin revealed a significantly higher number of nerve fibres expressing GAP-43 in both the acute and chronic stages of AIA. Importantly, the skin of arthritic rats in these regions did not contain a higher density of CD11b and CD3 immune cells than these regions in control rats. However, the lumbar DRGs of both the inflamed and noninflamed sides did exhibit a pronounced invasion of macrophages in the acute stage of inflammation.

GAP-43 has received considerable interest in basic as well as clinical neuroscience research because significant changes of GAP-43–positive nerve fibres were identified in human disease states. A reduction of IENF density is considered a hallmark of classical small-fibre peripheral neuropathy. In patients with neuropathy in the course of type 2 diabetes, IENF density was significantly lower than in controls [30, 31], corresponding to early dysfunction of sensory small-diameter nerve fibres and to depletion of SP and nerve growth factor (NGF) in the skin [32]. The IENF density was also reduced in patients with fibromyalgia concomitant with signs of impaired small-fibre function [33]. Interestingly, changes of IENF density are often associated with alterations of GAP-43. Thus, the reduction of IENF density in both type 2 diabetes [30] and fibromyalgia [33] is accompanied by a reduction of GAP-43. GAP-43–immunoreactive IENFs were also decreased in highly inflamed psoriasis skin areas [34], whereas a significant increase of GAP-43–positive IENFs was shown in human allergic contact dermatitis in eczematous skin compared with control skin [35, 36]. Thus, alterations in the expression of GAP-43 are found in different disease conditions, inviting discussion of the role of GAP-43.

GAP-43 is a membrane protein associated with axonal development, axonal regeneration, remodelling, and neuronal plasticity [22, 37]. It is highly expressed in the central nervous system (CNS) during development, assisting neuronal path finding and branching until mature synapses are formed. In some CNS regions, GAP-43 is expressed throughout adulthood, and it is thought to be involved in processes such as memory formation [38]. GAP-43 immunoreactivity was also found in a subset of PGP 9.5–immunoreactive sensory and autonomic fibres in the dermis and epidermis and around sweat glands, hair follicles, and blood vessels [22, 39]. GAP-43 is consistently expressed in these nerve fibres [22], suggesting that a proportion of these peripheral nerve fibres are in a state of restructuring. GAP-43 is upregulated after nerve injury, which is followed by regrowth of the injured nerve, and the upregulation is thought to be triggered by the interruption of the retrograde axonal transport [40, 41]. GAP-43 is critically involved in nerve regrowth because depletion of GAP-43 in neuronal growth cones leads to deficient spreading and branching of nerve fibres [42], whereas overexpression of GAP-43 induces spontaneous nerve sprouting and a highly potentiated sprouting response with branching after nerve lesions in transgenic mice [43].

Whether alterations of GAP-43 are per se (and causally) associated with sensory changes such as pain or sensory abnormalities is currently unknown. In clinical studies, researchers often use changes in the expression of GAP-43 as an indicator of neuronal changes, mostly as an indicator of regeneration or regenerative potential. However, researchers in most studies have not reported whether patients had symptoms such as pain or sensory abnormalities. Interestingly, investigators in one study reported that patients with diabetic neuropathy and pain had higher GAP-43/PGP 9.5 ratios than patients with diabetic neuropathy without pain [44]. However, firm conclusions on the role of GAP-43 in sensory function are premature.

The present data show some aspects related to this problem. First, in the present experiments, the density of GAP-43–positive IENFs per se did not correlate to hyperalgesia, because at day 3 of AIA the numbers of GAP-43–positive IENFs were scattered from the normal range to enhanced levels but the drop of pain thresholds was pronounced in all animals. Furthermore, at day 21, weight-bearing and primary hyperalgesia at the knee were clearly reversed; yet, all rats exhibited an increase of GAP-43–positive IENFs in the ipsilateral paw at this time point. This suggests that the reversal from a significant pain state in the deep tissue may be associated with an increase of GAP-43–positive IENFs (at least in some areas) (see Fig. 5). However, Fig. 1d shows a persistent reduction of the mechanical withdrawal thresholds upon von Frey hair stimulation at the ipsilateral paw, indicating persistence of secondary (cutaneous) hyperalgesia. Although this reduction was not statistically significant in the present study, we found a statistically significant persistent thermal hyperalgesia at the ipsilateral paw at day 21 in previous studies [4, 29]. We did not test for thermal hyperalgesia in the present study (to avoid any damage to the skin), and therefore we have no information on the relationship between thermal hyperalgesia and GAP-43 expression. However, in the AIA model, thermal hyperalgesia at the paw develops quickly in parallel to mechanical hyperalgesia [3, 4, 29], and thus neither mechanical nor thermal hyperalgesia per se seems to be strictly correlated with changes of GAP-43 expression.

It is important to note that the behavioural changes in experimental arthritis models correspond to observations in humans. Rats with unilateral arthritis in the knee joint also exhibit local mechanical hyperalgesia at the contralateral healthy knee joint, and they show enhanced sensitivity to mechanical and thermal stimuli at the hind paws [3, 4]. Widespread pain was also noted in bilateral experimental arthritis [45, 46]. Correspondingly, widespread pain was documented in patients with arthritis [1, 2]. Moreover, changes in skin sensation or cutaneous nociceptor function in arthritis were also reported in humans. Capsaicin-induced cutaneous vasodilation (flare) and hyperalgesia were increased in patients with rheumatoid arthritis and fibromyalgia [47–50], suggesting neuronal changes in areas of secondary hyperalgesia. The studies in patients match observations in animal experiments, which showed changes of nociceptive thresholds in areas remote from the inflamed joint. Such phenomena are usually explained by central mechanisms, but the present study indicates that peripheral changes should also be considered.

Whether humans with rheumatoid arthritis show enhanced GAP-43 expression has not been explored, to our knowledge. However, patients with rheumatoid arthritis do not have significantly altered IENF densities compared with healthy subjects [51]. This data is concordant with our findings in AIA rats, where IENF densities determined with primary antibodies against PGP 9.5 did not change either. Interestingly, patients with systemic lupus erythematosus and primary Sjögren syndrome presented with significantly decreased IENF densities compared with control subjects [51].

Although the sensory consequences of altered GAP-43 expression are unclear, the consensus based on many studies is that neuropathy or local inflammatory changes may be associated with changes in GAP-43 expression and that an upregulation of GAP-43 indicates a regeneration of nerve fibres, whereas a reduction of GAP-43 is associated with a loss of regenerating nerve fibres. The novel finding of the present study is that the GAP-43 expression in nerve fibres was increased in skin remote from an inflamed joint. The present findings are the first, to our knowledge, to indicate that IENFs remote from the joint show changes in arthritis.

The question regarding mechanisms which might be involved in the upregulation of GAP-43 in nerve fibres following arthritis in the skin remote from the joint is intriguing. Our data indicate that this process started within the first 3 days of AIA and persisted in some skin regions up to 21 days. In the AIA model, lumbar DRG neurons do not express activating transcription factor 3, a marker for neuronal damage, at the acute or chronic stage [20]. Thus, there is no evidence for a neuropathic process. CD11b- and CD3-immunoreactive cells in the ipsilateral rat paw were found at a similar density in the dermis of control rats, rats with acute AIA, and rats with chronic AIA. Thus, we did not obtain any evidence for an inflammatory process in the paw pad remote from the inflamed knee joint. Preliminary data do not indicate that the upregulation of GAP-43 is correlated to NGF in the paw (unpublished data from E Jochmann, MK Boettger, P Anand, HG Schaible, 2015).

In the present study, we observed, as in two previous studies, a bilateral invasion of macrophages into the lumbar DRGs of both the inflamed and noninflamed sides [20, 52]. This invasion was seen mainly at the acute stage of AIA [20]. Careful examination of the invading macrophages showed that they resembled the tumour necrosis factor–activated type of macrophage, a subtype of M1 macrophages. These macrophages do not express inducible nitric oxide synthase and do not destroy neurons, but they do release mediators such as interleukin (IL)-6 and are able to stimulate DRG neurons [52]. An important aspect is that these macrophages seem to be distributed throughout the DRGs [20, 52]. Thus, they may influence the cell bodies of DRG neurons supplying different target tissues in the leg. This observation supports the conclusion that localized arthritis may affect sensory neurons beyond those supplying the inflamed joint. In fact, the upregulation of some receptors, such as IL-1 receptor type I, during AIA is observed in a large proportion of DRG neurons in the lumbar DRGs [4].

Whether the invasion of macrophages into the DRGs is linked to enhanced GAP-43 expression is unknown. The data shown in Fig. 9 allow at least some speculation. The invasion of macrophages was strongly correlated with changes in weight-bearing and primary hyperalgesia at the inflamed knee joint in rats with AIA. At day 21 of AIA, primary hyperalgesia and asymmetric weight-bearing were reversed and macrophage invasion disappeared. However, secondary hyperalgesia persisted at this point and GAP-43 expression was increased. It may be speculated, therefore, that the invasion of macrophages into the DRGs triggers changes in the neurons which lead to an upregulation of GAP-43 and the persistence of secondary hyperalgesia remote from the joint.

## Conclusions

On the basis of the present study, we report the novel observation that changes of innervation in arthritis models occur not only in the inflamed joint but also in healthy skin remote from the joint. Because GAP-43 is thought to indicate processes of structural neuronal plasticity, the present findings suggest that arthritis is associated with neuronal changes in skin innervation remote from the inflamed joint. Although the precise role of enhanced GAP-43 expression in mechanisms of pain is not known, the data show that localized immune-mediated arthritis may cause more widespread neuronal alterations in the peripheral nervous system. Such neuronal changes may contribute to long-lasting secondary hyperalgesia in the skin remote from the inflamed joint. Our findings should stimulate research into the regulation and functional importance of GAP-43 and regenerating nerve fibres in arthritis and chronic, widespread pain.

## Abbreviations

AIA: Antigen-induced arthritis
CD: Cluster of differentiation
CGRP: Calcitonin gene–related peptide
CNS: Central nervous system
DRG: Dorsal root ganglion
ED1: Anti-CD68 antibody (clone ED1)
EFNS: European Federation of Neurological Societies
GAP-43: Growth-associated protein 43
IENF: Intraepidermal nerve fibre
IL: Interleukin
mBSA: Methylated bovine serum albumin
NGF: Nerve growth factor
PBS: Phosphate-buffered saline
PGP 9.5: Protein gene product 9.5
SD: Standard deviation
SP: Substance P
TRPV1: Transient receptor potential vanilloid 1

## References

[1] Arendt-Nielsen L, Nie H, Laursen MB, Laursen BS, Madeleine P, Simonsen OH (2010). Sensitization in patients with painful knee osteoarthritis. Pain.

[2] Hendiani JA, Westlund KN, Lawand N, Goel N, Lisse J, McNearney T (2003). Mechanical sensation and pain thresholds in patients with chronic arthropathies. J Pain.

[3] Boettger MK, Hensellek S, Richter F, Gajda M, Stöckigt R, von Banchet GS (2008). Antinociceptive effects of tumor necrosis factor alpha neutralization in a rat model of antigen-induced arthritis: evidence of a neuronal target. Arthritis Rheum.

[4] Ebbinghaus M, Uhlig B, Richter F, von Banchet GS, Gajda M, Bräuer R (2012). The role of interleukin-1β in arthritic pain: main involvement in thermal but not in mechanical hyperalgesia in rat antigen-induced arthritis. Arthritis Rheum.

[5] Phillips K, Clauw DJ (2013). Central pain mechanisms in the rheumatic diseases. Arthritis Rheum.

[6] Woolf CJ, Salter MW (2000). Neuronal plasticity: increasing the gain in pain. Science.

[7] Gwilym SE, Keltner JR, Warnaby CE, Carr AJ, Chizh B, Chessell I (2009). Psychophysical and functional imaging evidence supporting the presence of central sensitization in a cohort of osteoarthritis patients. Arthritis Rheum.

[8] Bushnell MC, Ceko M, Low LA (2013). Cognitive and emotional control of pain and its disruption in chronic pain. Nature Rev Neurosci.

[9] Kosek E, Ordeberg G (2000). Lack of pressure pain modulation by heterotopic noxious conditioning stimulation in patients with painful osteoarthritis before, but not following surgical pain relief. Pain.

[10] Woolf CJ (1983). Evidence for a central component of post-injury pain hypersensitivity. Nature.

[11] Neugebauer V, Lücke T, Schaible HG (1993). *N*-methyl-d-aspartate (NMDA) and non-NMDA receptor antagonists block the hyperexcitability of dorsal horn neurons during development of acute arthritis in rat’s knee joint. J Neurophysiol.

[12] Grönblad M, Konttinen YT, Korkola O, Liesi P, Hukkanen M, Polak JM (1988). Neuropeptides in synovium of patients with rheumatoid arthritis and osteoarthritis. J Rheumatol.

[13] Hukkanen M, Grönblad M, Rees R, Konttinen YT, Gibson SJ, Hietanen J (1991). Regional distribution of mast cells and peptide containing nerves in normal and adjuvant arthritic rat synovium. J Rheumatol.

[14] Konttinen YT, Rees R, Hukkanen M, Grönblad M, Tolvanen E, Gibson SJ (1990). Nerves in inflammatory synovium: immunohistochemical observations on the adjuvant arthritic rat model. J Rheumatol.

[15] Mapp PI, Kidd BL, Gibson SJ, Terry JM, Revell PA, Ibrahim NB (1990). Substance P-, calcitonin gene-related peptide- and C-flanking peptide of neuropeptide Y-immunoreactive fibres are present in normal synovium but depleted in patients with rheumatoid arthritis. Neuroscience.

[16] da Silva JA P, Carmo-Fonseca M (1990). Peptide containing nerves in human synovium: immunohistochemical evidence for decreased innervation in rheumatoid arthritis. J Rheumatol.

[17] Miller LE, Jüsten HP, Schölmerich J, Straub RH (2000). The loss of sympathetic nerve fibers in the synovial tissue of patients with rheumatoid arthritis is accompanied by increased norepinephrine release from synovial macrophages. FASEB J.

[18] Eitner A, Pester J, Nietzsche S, Hofmann GO, Schaible HG (2013). The innervation of synovium of human osteoarthritic joints in comparison with normal rat and sheep synovium. Osteoarthritis Cartilage.

[19] Jimenez-Andrade JM, Mantyh PW (2012). Sensory and sympathetic nerve fibers undergo sprouting and neuroma formation in the painful arthritic joint of geriatric mice. Arthritis Res Ther.

[20] Segond von Banchet G, Boettger MK, Fischer N, Gajda M, Bräuer R, Schaible H-G (2009). Experimental arthritis causes tumor necrosis factor-alpha-dependent infiltration of macrophages into rat dorsal root ganglia which correlates with pain-related behavior. Pain.

[21] Julius D (2013). TRP channels and pain. Annu Rev Cell Dev Biol.

[22] Fantini F, Johansson O (1992). Expression of growth-associated protein 43 and nerve growth factor receptor in human skin: a comparative immunohistochemical investigation. J Invest Dermatol.

[23] Boettger MK, Leuchtweis J, Kümmel D, Gajda M, Bräuer R, Schaible HG (2010). Differential effects of locally and systemically administered soluble glycoprotein 130 on pain and inflammation in experimental arthritis. Arthritis Res Ther.

[24] Leuchtweis J, Imhof AK, Montechiaro F, Schaible HG, Boettger MK (2010). Validation of the digital pressure application measurement (PAM) device for detection of primary hyperalgesia in rat and mouse antigen-induced knee joint arthritis. Methods Find Exp Clin Pharmacol.

[25] Facer P, Mathur R, Pandya SS, Ladiwala U, Singhal BS, Anand P (1998). Correlation of quantitative tests of nerve and target organ dysfunction with skin immunohistology in leprosy. Brain.

[26] Curtis R, Hardy R, Reynolds R, Spruce BA, Wilkin GP (1991). Down-regulation of GAP43 during oligodendrocyte development and lack of expression by astrocytes in vivo: implications for macroglial differentiation. Eur J Neurosci.

[27] Shu SY, Ju G, Fan LZ (1988). The glucose oxidase-DAB-nickel method in peroxidase histochemistry of the nervous system. Neurosci Lett.

[28] Lauria G, Cornblath DR, Johansson O, McArthur JC, Mellgren SI, Nolano M (2005). EFNS guidelines on the use of skin biopsy in the diagnosis of peripheral neuropathy. Eur J Neurol.

[29] Segond von Banchet G, König C, Patzer J, Eitner A, Leuchtweis J, Ebbinghaus M, et al. Longlasting activation of the transcription factor CREB in sensory neurons by interleukin-1β during antigen-induced arthritis in rat: a mechanism of persistent arthritic pain? Arthritis Rheum. In press. doi:10.1002/art.39445. 10.1002/art.39445 26473326

[30] Bursova S, Dubovy P, Vlckova-Moravcova E, Nemec M, Klusakova I, Belobradkova J (2012). Expression of growth-associated protein 43 in the skin nerve fibers of patients with type 2 diabetes mellitus. J Neurol Sci.

[31] Narayanaswamy H, Facer P, Misra VP, Timmers M, Byttebier G, Meert T (2012). A longitudinal study of sensory biomarkers of progression in patients with diabetic peripheral neuropathy using skin biopsies. J Clin Neurosci.

[32] Anand P, Terenghi G, Warner G, Kopelman P, Williams-Chestnut RE, Sinicropi DV (1996). The role of endogenous nerve growth factor in human diabetic neuropathy. Nat Med.

[33] Üçeyler N, Zeller D, Kahn AK, Kewenig S, Kittel-Schneider S, Schmid A (2013). Small fibre pathology in patients with fibromyalgia syndrome. Brain.

[34] El-Nour H, Santos A, Nordin M, Jonsson P, Svensson M, Nordlind K (2009). Neuronal changes in psoriasis exacerbation. J Eur Acad Dermatol Venereol.

[35] El-Nour H, Lundeberg L, Al-Tawil R, Granlund A, Lonne-Rahm SB, Nordlind K (2006). Upregulation of the axonal growth and the expression of substance P and its NK1 receptor in human allergic contact dermatitis. Immunopharmacol Immunotoxicol.

[36] Altawil R, Lyström J, El-Nour H (2012). Kinetics of neuronal contribution during the development of a contact allergic reaction. Arch Dermatol Res.

[37] Denny JB (2006). Molecular mechanisms, biological actions, and neuropharmacology of the growth-associated protein GAP-43. Curr Neuropharmacol.

[38] Benowitz LI, Perrone-Bizzozero NI, Finklestein SP, Bird ED (1989). Localization of the growth-associated phosphoprotein GAP-43 (B-50, F1) in the human cerebral cortex. J Neurosci.

[39] Verze L, Viglietti-Panzica C, Maurizo S, Sica M, Panzica G (2003). Distribution of GAP-43 nerve fibers in the skin of the adult human hand. Anat Record.

[40] Verhaagen J, Edwards PM, Veldman H, Jennekens FGI, Gispen WH (1988). Light- and electron-microscopical study of phosphoprotein B-50 following denervation and reinnervation of the rat soleus muscle. J Neurosci.

[41] Woolf CJ, Reynolds ML, Molander C, O’Brien C, Lindsay RM, Benowitz LI (1990). The growth-associated protein gap-43 appears in dorsal root ganglion cells and in the dorsal horn of the rat spinal cord following peripheral nerve injury. Neuroscience.

[42] Aigner L, Caroni P (1995). Absence of persistent spreading, branching, and adhesion in GAP-43-depleted growth cones. J Cell Biol.

[43] Aigner L, Arber S, Kapfhammer JP, Laux T, Schneider C, Botteri F (1995). Overexpression of the neural growth-associated protein GAP-43 induces nerve sprouting in the adult nervous system of transgenic mice. Cell.

[44] Cheng HT, Dauch JR, Porzio MT, Yanik BM, Hsieh W, Smith AG (2013). Increased axonal regeneration and swellings in intraepidermal nerve fibers characterize painful phenotype of diabetic neuropathy. J Pain.

[45] Christianson CA, Corr M, Firestein GS, Mobargha A, Yaksh TL, Svensson CI (2010). Characterization of the acute and persistent pain state present in K/BxN serum transfer arthritis. Pain.

[46] Inglis JJ, Notley CA, Essex D, Wilson AW, Feldmann M, Anand P (2007). Collagen-induced arthritis as a model of hyperalgesia: functional and cellular analysis of the analgesic actions of tumor necrosis factor blockade. Arthritis Rheum.

[47] Jolliffe VA, Anand P, Kidd BL (1995). Assessment of cutaneous sensory and autonomic axon reflexes in rheumatoid arthritis. Ann Rheum Dis.

[48] Morris VH, Cruwys SC, Kidd BL (1997). Characterisation of capsaicin-induced mechanical hyperalgesia as a marker for altered nociceptive processing in patients with rheumatoid arthritis. Pain.

[49] Morris V, Cruwys S, Kidd B (1998). Increased capsaicin-induced secondary hyperalgesia as a marker of abnormal sensory activity in patients with fibromyalgia. Neurosci Lett.

[50] Lautenbacher S, Rollman GB, McGain GA (1994). Multi-method assessment of experimental and clinical pain in patients with fibromyalgia. Pain.

[51] Goransson LG, Brun JG, Harboe E, Mellgren SI, Omdal R (2006). Intraepidermal nerve fiber densities in chronic inflammatory autoimmune diseases. Arch Neurol.

[52] Massier J, Eitner A, Segond von Banchet G, Schaible HG (2015). Effects of differently activated rodent macrophages on sensory neurons: implications for arthritis pain. Arthritis Rheumatol.

